# Health Information–Seeking Behavior of Seniors Who Use the Internet: A Survey

**DOI:** 10.2196/jmir.3749

**Published:** 2015-01-08

**Authors:** Stephanie Medlock, Saeid Eslami, Marjan Askari, Derk L Arts, Danielle Sent, Sophia E de Rooij, Ameen Abu-Hanna

**Affiliations:** ^1^Academic Medical CenterDepatment of Medical InformaticsUniversity of AmsterdamAmsterdamNetherlands; ^2^Pharmaceutical Research CenterSchool of PharmacyMashhad University of Medical SciencesMashhadIslamic Republic Of Iran; ^3^Academic Medical CenterDepatment of General PracticeUniversity of AmsterdamAmsterdamNetherlands; ^4^Academic Medical CenterDepatment of Internal Medicine, Section of Geriatric MedicineUniversity of AmsterdamAmsterdamNetherlands; ^5^Department of Geriatric MedicineUniversity Medical Centre GroningenGroningenNetherlands

**Keywords:** aged, aged, 80 and over, information-seeking behavior, Internet, patient education, empowerment

## Abstract

**Background:**

The Internet is viewed as an important source for health information and a medium for patient empowerment. However, little is known about how seniors use the Internet in relation to other sources for health information.

**Objective:**

The aim was to determine which information resources seniors who use the Internet use and trust for health information, which sources are preferred, and which sources are used by seniors for different information needs.

**Methods:**

Questions from published surveys were selected based on their relevance to the study objectives. The Autonomy Preference Index was used to assess information needs and preferences for involvement in health decisions. Invitation to participate in this online survey was sent to the email list of a local senior organization (298 addresses) in the Netherlands.

**Results:**

There were 118 respondents with a median age of 72 years (IQR 67-78 years). Health professionals, pharmacists, and the Internet were the most commonly used and trusted sources of health information. Leaflets, television, newspapers, and health magazines were also important sources. Respondents who reported higher use of the Internet also reported higher use of other sources (*P*<.001). Use of health professionals, pharmacists, leaflets, telephone, television, and radio were not significantly different; use of all other resources was significantly higher in frequent Internet users. When in need of health information, preferred sources were the Internet (46/105, 43.8%), other sources (eg, magazines 38/105, 36.2%), health professionals (18/105, 17.1%), and no information seeking (3/105, 2.8%). Of the 51/107 respondents who indicated that they had sought health information in the last 12 months, 43 sought it after an appointment, 23 were preparing for an appointment, and 20 were deciding if an appointment was needed. The source used varied by the type of information sought. The Internet was used most often for symptoms (27/42, 64%), prognosis (21/31, 68%), and treatment options (23/41, 62%), whereas health professionals were asked for additional information on medications (20/36, 56%), side effects (17/36, 47%), coping (17/31, 55%), practical care (12/14, 86%), and nutrition/exercise (18/30, 60%).

**Conclusions:**

For these seniors who use the Internet, the Internet was a preferred source of health information. Seniors who report higher use of the Internet also report higher use of other information resources and were also the primary consumers of paper-based resources. Respondents most frequently searched for health information after an appointment rather than to prepare for an appointment. Resources used varied by health topic. Future research should seek to confirm these findings in a general elderly population, investigate how seniors seek and understand information on the Internet, and investigate how to reach seniors who prefer not to use the Internet for health information.

## Introduction

Patient empowerment is defined by the World Health Organization as “a process through which people gain greater control over decisions and actions affecting their health” [1]. Patient empowerment is viewed as both a practical and moral necessity: a means to change health-related behaviors, to control costs, improve the quality of care, ensure continuity of care, improve the patient experience, and enable shared decision making so that patients can participate more fully in decisions about their own health and health care [2]. An informed decision can be defined as one that is based on relevant, high-quality information and reflects the decision maker’s values [3]. Thus, access to high-quality information is a prerequisite for shared decision making.

The Internet is an important resource for health information [4]. A recent survey of the general Dutch population showed that the Internet is widely used for health information [5]. Seniors are the fastest-growing group of Internet users in the Netherlands [6] and Europe [7]. In a national survey in the Netherlands in 2012, 81% of people aged 65-75 years used the Internet and 54% used the Internet for health information. However, this does not tell us whether the Internet is used frequently or infrequently, why and how the Internet was used for gathering health information, or whether seniors feel they can trust the information they find there. It also does not address how use of the Internet compares to other sources of health information and whether seniors would prefer to get health information from some other source. If we know more about why and how seniors seek health information, we can more efficiently and effectively empower seniors by using the right information resource at the right time. Thus, we sought to learn what resources seniors who use the Internet use and trust for health care information, their preferences regarding sources of information, and the resources used in relation to the timing and types of information sought.

## Methods

### Survey Development

Questions from the survey “e-Health and the Internet: How Seniors Use the Internet for Health Information” [8] were expanded with questions from other published surveys on health information seeking [4,9-15]. The questions were selected based on relevance to our study questions: which health information resources seniors use and trust, and whether they use different sources for different types of information. The questions were reviewed by 3 experts in medical informatics (AA, SE, and DS) and a geriatrician (SdR). To assess respondents’ preferences for information, we used the questions from the Autonomy Preference Index (API) [16] reported to have good reliability in a German study population [17]. The API is a validated survey to assess the desire for information and preferences for involvement in medical decision making. It consists of two 100-point scales, Decision Making and Information Needs, where 0 represents the minimum possible score and 100 the maximum [17]. References to the source of each question are included with the annotated survey instrument in Multimedia Appendix 1. All survey questions were forward- and back-translated to Dutch (DS and SM). Before deployment, a geriatrician (SdR) and the head of the local senior organization filled in the resulting survey and were asked to give feedback on whether the questions were understandable for seniors, whether any questions seemed out of place, and the time required to fill in the survey. Their feedback was incorporated into the survey by 2 of the researchers (SM and SE).

### Survey Deployment

Members of a local organization for seniors (Protestants Christelijke Ouderen Bond) affiliated with a national organization who had an email address listed with their organization were contacted via email. One reminder email was sent 2 weeks later. The emails contained a brief description of the survey and research project, and a link to fill in the survey online using a commercial survey site (Survey Gizmo). The survey was presented with 1 page per section, with an introduction on each page explaining the purpose of the questions. The introduction to the first section included the information that collection and analysis of survey data were anonymous.

The Medical Ethics Committee of the Academic Medical Center of the University of Amsterdam determined that this study was exempt from the need for approval under the Netherlands Medical Research Involving Human Subjects Act.

### Analysis

#### Statistical Analyses

Differences between rating scales were calculated using the Wilcoxon rank sum test. All other associations between variables were assessed using linear regression, and the result reported as the standardized coefficient (β) with a confidence interval (CI). In the analyses of use of Internet related to use of other sources of health information, respondents who reported using the Internet for health information “a lot” or “a fair amount” were compared to the group that used the Internet “a little” or “not at all.” The *P* value was adjusted with the false discovery rate (FDR) correction whenever >5 hypotheses were tested on the same dataset, using an overall significance level and accepted q-value of .05 (indicated by *P*_adj_). All analyses were performed using R 3.1.0 [18].

#### Duplicate and Missing Responses

It was possible for the same respondent to fill in the survey multiple times. If 2 responses originated from the same Internet protocol address and had identical demographics, then responses were considered to have originated from the same person. In those cases, numeric responses were the mean of the 2 responses; in nominal responses, the most recent answer was used. Respondents were allowed to skip questions; therefore, we analyzed each question with n equal to the number of responses to that question. When the respondent filled in a source that had been used when searching for health information, but failed to check “yes” indicating that they had searched for that information, we assigned a “yes” response by inference.

#### Socioeconomic Status

Socioeconomic status (SES) was derived from the 4-digit postcode based on data from The Netherlands Institute for Social Research [19]. The number represents a combination of average educational level, income, and market position of the neighborhood. Zero represents the national average and national scores per postcode range from -3.65 to 6.04.

## Results

### Survey Instrument

The final survey instrument (given in Multimedia Appendix 1) consisted of 6 sections: demographics, the API, use and trust of information sources, the timing and subjects of information sought in the last 12 months, perceived need for additional information, and the consequences of information seeking. The evaluation by the geriatrician and the head of the senior organization suggested minor changes to the presentation, indicated that the survey was understandable for older people, and indicated that the survey required 30 minutes to complete.

### Survey Results

#### Overview

The invitation to participate was sent to all 298 email addresses available from the 670 members of the organization and responses were collected from October 25 to November 25, 2011. The site received 184 visits during this time, of which 130 resulted in at least 1 response. Of these, 11 were judged to be from an earlier respondent according to our matching criteria and were combined with 10 previous responses, resulting in a total of 118 responses and a maximum of 173 unique visitors (minimum of 68% participation rate), with a completeness rate of 103/118 (87.3%). A total of 913/9676 (9.44%) of responses were missing or 475/8446 (5.62%) of responses in completed surveys. An additional 65/9676 (0.67%) of responses were imputed. The number of respondents per section is given in the results for each section.

#### Demographics and the Autonomy Preference Index

The demographics of respondents are given in Table 1. The median age of respondents was 72 years, with a range of 49-94 years (IQR 67-78). Of these, 88.9% (105/118) were aged 65 years or older. The respondents were 56.0% women (65/116), primarily with a high school–equivalent education (61/117, 52.1%), of Dutch ethnicity (108/114, 94.7%), and in good health (69/117, 59.0%). In all, 18/116 (15.5%) were the caretaker for someone with a serious health condition and all but 1 (116/117, 99.1%) were community-dwelling. The quality of health services were rated highly: 106/116 (91.4%) felt they could get an appointment as quickly as they wanted and the overall health care received a median score of 8/10 (range 0-10, IQR 7-10). Four respondents reported that they had assistance in filling in the survey.

The median score on the API Involvement in Decision Making scale was 58 (IQR 42-67) and the median score on the API Information Needs scale was 71 (IQR 66-90). The Information Needs score was not significantly associated with age, gender, SES, education, health status, or caretaker status; however, a higher score on the Decision Making scale was associated with both lower age (β=–0.07, 95% CI –0.12 to –0.03, *P=*.002) and higher educational level (β=0.37, 95% CI 0.08-0.65, *P=*.01).

**Table 1:** Demographics of respondents in the survey (N=118).

Item		Participants
Age (years), mean (IQR)		72 (67-78)
Gender (female; N=116), n (%)		65 (56.0)
Socioeconomic status^a^ (N=116), median (IQR)		0.27 (0.16-0.27)
**Educational level (N=117), n (%)**		
	Primary school	2 (1.7)
	Some high school	14 (12.0)
	Vocational/technical school	5 (4.2)
	High school	61 (52.1)
	Vocational school	10 (8.5)
	University	25 (21.4)
**Country of birth (N=114), n (%)**		
	Netherlands	108 (94.7)
	Other	6 (5.1)
Is a primary caretaker (N=116), n (%)		18 (15.5)
**Health status (N=117), n (%)**		
	Very good	7 (6.0)
	Good	69 (59.0)
	Fair	37 (31.6)
	Poor	4 (3.4)
	Very poor	0 (0.0)
**Perceived access to care (“I can see my primary care practitioner as soon as I want” N=116), n (%)**		
	Strongly agree	28 (24.1)
	Agree	78 (67.2)
	Disagree	9 (7.8)
	Strongly disagree	1 (0.9)
**Rating of overall quality of care (0-10 scale, N=112), median (IQR)**		8 (7-10)
**Assistance filling in survey (N=94), n (%)**		
	Yes	4 (4)
	No	90 (96)

^a^ Based on postcode. National average=0.

#### Use and Trust of Sources of Health Information

Respondents’ self-reported use and trust in various sources of health information are given in Table 2. Health professionals, pharmacists, and the Internet were the most commonly used, with respectively 61/115 (53.0%), 59/111 (53.1%), and 60/113 (53.1%) of respondents indicating that they used each source “a lot” or “a fair amount”. Health professionals, pharmacists, and the Internet were also the 3 most-trusted sources of health information with respectively 75.5% (80/106), 72.9% (78/107), and 40.4% (42/104) of respondents indicating that they trust these sources “a lot” or “a fair amount”.

Respondents were also asked how much they trust health information from each resource. Generally, trust closely tracked use, implying that the underlying construct is the same or highly correlated for most resources. However, trust in the resource was significantly higher than use of the resource for health professionals and pharmacists, and significantly lower for television and newspapers (Table 2).

**Table 2:** Use and trust of health information sources and use of non-Internet health information sources by Internet use. Respondents were allowed to skip questions; therefore, the n/N is reported per question. *P* values are corrected for multiple testing.

Information resource	Use and trust of information sources: all respondents			Use of information sources a lot/a fair amount by Internet use		
	Use resource a lot/a fair amount (median 23%), n/N (%)	Trust resource a lot/a fair amount (median 25%), n/N (%)	Difference in use and trust, P_adj_	Use Internet a lot/a fair amount (N=60; median 30%), n/N (%)	Use the Internet a little/not at all (N=53; median 9%), n/N (%)	Difference in use of other resources, P_adj_
Face-to-face contact with a health professional	61/115 (53.0)	80/106 (75.5)	.002	28/59 (47)	27/53 (51)	.10
Pharmacists	59/111 (53.2)	78/107 (72.9)	.02	35/59 (59)	22/49 (45)	.16
Leaflets at the doctor’s office	47/115 (40.9)	37/105 (35.2)	.43	26/59 (44)	20/53 (38)	.24
Telephone helpline	14/113 (12.4)	17/106 (16.0)	.23	11/58 (19)	3/53 (6)	.07
Television	35/115 (30.4)	14/105 (13.3)	.001	21/59 (36)	13/53 (24)	.06
Radio	14/115 (12.2)	11/106 (10.4)	.23	8/59 (14)	5/53 (9)	.13
Newspapers	40/114 (35.1)	21/105 (20.0)	<.001	27/59 (46)	12/53 (23)	.007
Health magazines	36/114 (31.6)	23/107 (21.5)	.29	26/59 (44)	9/52 (17)	.004
Other magazines	20/112 (17.9)	10/103 (9.7)	.12	15/56 (27)	4/53 (8)	.007
Family and friends	27/114 (23.7)	20/105 (19.0)	.29	19/58 (33)	6/53 (11)	.004
Church/religious group	2/112 (1.8)	1/101 (1.0)	.43	2/58 (3)	0/52 (0)	.05
Courses and lectures	11/111 (9.9)	16/105 (15.2)	.44	11/57 (19)	0/52 (0)	.07
Internet	60/113 (53.1)	42/104 (40.4)	.12	—	—	—
Self-help/support group	17/110 (15.5)	17/105 (16.2)	.43	14/57 (25)	2/52 (4)	.004
Books/encyclopedias	24/112 (21.4)	20/101 (19.8)	.65	19/58 (33)	4/52 (8)	.004
The library	14/111 (12.6)	11/103 (10.7)	.79	12/57 (21)	2/53 (4)	.02

Use of the Internet for health information decreased slightly with age (β=–0.02 per year, 95% CI –0.05 to –0.001, *P=*.03), but was not associated with gender, education, health status, or caretaker status. The association with age did not persist when the analysis was restricted to respondents aged 65 years and older (n=102). Use of other (non-Internet) information sources also was not associated with age, gender, education, health status, or caretaker status.

Overall use of information sources was not associated with either the Decision Making or Information Needs scales of the API (Decision Making: β=–0.01, 95% CI –0.06 to –0.04, *P=*.65; Information Needs: β=0.06, 95% CI –0.02 to 0.14, *P=*.13), nor was use of the Internet for health information (Decision Making: β=0.21, 95% CI –0.18 to 0.61, *P=*.29; Information Needs: β=0.39, 95% CI –0.22 to 1.0, *P=*.23). However, higher use of other (non-Internet) information sources was associated with higher use of the Internet (β=0.06, CI 0.04-0.08, *P*<.001). Use of health professionals, pharmacists, leaflets, telephone helplines, television, and radio were not significantly different; use of all other resources was significantly higher for Internet users (Table 2).

When asked which resources are preferred when they have a need for health information, 46/105 (43.8%) respondents indicated that they preferred the Internet. An additional 38/105 (36.2%) indicated that they prefer resources other than the Internet and 18/105 (17.1%) indicated that they prefer to only ask health professionals. The remaining 3/105 (2.9%) said that they have no need for additional health information. For the 92 respondents aged 65 years and older, responses were similar: 42 (46%) preferred Internet, 31 (34%) preferred other resources, 16 (15%) preferred health professionals, and 3 (3%) indicated that they had no need for additional information. In a post hoc sensitivity analysis, there was no significant difference between the proportions of respondents that preferred the Internet in the age categories of <65 years (4/13, 30%) and 65-75 years (22/54, 41%; *P*=.64), those aged 65-75 and >75 years (20/38, 53%; *P=*.53), or those <65 years and >75 years (*P=*.33).

#### Timing and Types of Information Sought

Of the 107 respondents completing the section on timing of information seeking, 51/107 (47.7%) indicated that they had sought health information in relation to an appointment with a health professional in the last 12 months (Table 3). Of these, 43/51 (84%) indicated that they sought information after an appointment. Far fewer had sought information to prepare for an appointment (23/51, 45%) or to decide if an appointment was needed (20/51, 39%). Reponses were similar for those aged 65 years and older (45/93 indicated they had sought health information in the last 12 months: 37/45, 82% after an appointment; 21/45, 46% to prepare for an appointment; and 19/45, 42% to decide if an appointment was needed). In all cases, the Internet was used more than other sources. Women were more likely than men to seek health information after an appointment (30 women and 13 men, β=–0.26, 95% CI –0.45 to –0.07, *P*_adj_ *=*.01); there were no other significant associations of the timing of information seeking with age or gender (see Multimedia Appendix 2).

Of the 107 respondents who completed the section on specific types of information sought, 70 (66%) indicated that they had sought health information on 1 or more of the listed subjects in the last 12 months (Table 4). The resources used varied widely by subject area. Respondents preferentially used the Internet when seeking additional information about symptoms (64%, 27/42), prognosis (68%, 21/31), and treatment options (62%, 23/37), but tended to ask health professionals for additional information about prescription medications (56%, 20/36) and side effects (47%, 17/36), practical care information (86%, 12/14), and nutrition/exercise advice (60%, 18/30). Advice from non–health professionals and paper-based resources were not commonly used, except for paper-based information about side effects (44%, 16/36). More women sought information in the last 12 months than men (women: 46/58; men: 24/49; β=–0.31, 95% CI –0.51 to –0.12, *P*_adj_ *=*.002). There were no gender differences in the types of information sought after correction for this and no association with age (see Multimedia Appendix 2).

**Table 3:** Timing and source of information seeking in relation to a doctor’s appointment.

Timing of information seeking	Sought information within last 12 months, n/N (%)	Source of information, n/N (%)			
		Asked a health professional	Asked someone else (eg, family or friends)	Looked on Internet	Information on paper
After an appointment	43/104 (41)	5/37 (14)	11/37 (30)	29/37 (78)	3/37 (8)
To prepare for an appointment	23/103 (22)	3/16 (19)	7/16 (44)	10/16 (63)	0/16 (0)
To decide if an appointment is needed	20/106 (19)	5/18 (28)	7/18 (39)	8/18 (44)	0/18 (0)

#### Need for Additional Health Information

One-third of respondents (34/104) indicated that they had a need for additional health information but did not know where to find it. Most (98/106) indicated that they did not have difficulty finding information in their own language. Nearly all respondents (101/106) indicated that they expect their doctor to provide them with all necessary information.

A total of 74 respondents reported seeking information on 1 or more subjects in the past 12 months, of which 63 specified a source for that information. Respondents were allowed to choose multiple sources of information.

**Table 4:** Subjects of information seeking.

Subject	Sought information within last 12 months, n/N (%)	Sources for health information on specified subject,^a^ n/N (%)			
		Asked a health professional	Asked someone else (eg, family or friends)	Looked on Internet	Information on paper
Look up symptoms to determine cause	46/105 (43.8)	18/42 (43)	7/42 (17)	27/42 (64)	4/42 (10)
Prognosis	34/104 (32.7)	16/31 (52)	6/31 (19)	21/31 (68)	3/31 (10)
Treatment options	41/102 (40.2)	20/37 (54)	2/37 (5)	23/37 (62)	5/37 (14)
Prescription medications	43/102 (42.2)	20/36 (56)	2/36 (6)	11/36 (31)	11/36 (31)
Side effects of treatment or medicines	45/100 (45.0)	17/36 (47)	2/36 (6)	8/36 (22)	16/36 (44)
Coping with an illness	33/101 (32.7)	17/31 (55)	6/31 (19)	17/31 (55)	4/31 (13)
Practical care information (eg, showering after a surgery)	18/102 (17.6)	12/14 (86)	1/14 (7)	2/14 (14)	2/14 (14)
Nutrition/exercise	40/103 (38.8)	18/30 (60)	5/30 (17)	13/30 (43)	6/30 (20)
Total (all topics)	74/106 (69.8)	151/430 (35.1)	56/430 (13.0)	169/430 (39.3)	54/430 (12.6)

^a^ Number specifying at least 1 source.

## Discussion

### Principal Results

Health professionals, pharmacists, and the Internet were the most used and the most trusted sources of health information in this group of seniors who use the Internet, although trust in pharmacists and other health professionals was higher than trust in the Internet. Responses on the API were not correlated with overall use of health information resources or with use of the Internet for health information. Use of the Internet was strongly correlated with use of other information resources. Respondents who reported using the Internet “a little” or “not at all” for health information reported using health professionals, pharmacists, leaflets, telephone information, TV, and radio approximately the same amount/extent as Internet users, but all other sources were used significantly more by Internet users. The Internet was also the most often preferred source for additional health information. Concerning the timing of seeking information, most respondents sought information after seeing a health professional, whereas only about half as many reported seeking information to prepare for a doctors’ visit or to decide if they needed to see a doctor. Different resources were used for different health information subjects: the Internet was predominantly used when searching for information on symptoms, prognosis, and treatment options, whereas health professionals were predominantly used for information on prescriptions, side effects, practical care information, and nutritional advice. One-third of respondents reported a need for more information that they did not know how to find. Nearly all respondents reported that they expect their health professional to provide all necessary information.

### Limitations

The main limitation of this study is the study sample. To avoid the confounding effect of the many possible barriers to Internet use in general, we chose to survey only people who already use the Internet and, thus, could potentially use it for health information. However, this limits the generalizability of our results because seniors who do not use the Internet were excluded. We anticipated a larger population of seniors who do not use the Internet for health information. For example, in a recent US study, younger adults were 3 times more likely to seek health information on the Internet than people older than age 65 [20]. However, only 18 seniors in our sample reported that they did not use the Internet for health information, limiting our analysis of the preferences of this population. Our survey found much higher use of the Internet for health information by seniors than the US survey from which the questions were drawn [8], which found only 21% of seniors had ever used the Internet for health information. However, only 31% of the seniors participating in the US survey had gone online or used email, implying that 68% of seniors in that study who used the Internet also used it for health information. A similar percentage of people aged 65-75 years used the Internet in the Netherlands in 2005 (35%) [6], the year of the US study, implying that the difference in this result between the original study and ours may be primarily because of increasing Internet use in both countries over time. More recent studies show higher rates of Internet use in older adults; for example, 53% in a recent US survey [21] and 45% of people aged 65-74 years in a 2007 Canadian survey (52% of which used it for health information) [22]. Our study is relatively small, with respondents from 1 senior organization, primarily from 1 geographic region of the Netherlands, who were fairly uniform in ethnic background. Thus, we were not able to investigate factors such as ethnicity or rural compared to urban residence, which may influence the use of the Internet for health information [23]. It is likely that some of our respondents knew one another and, although unlikely, it is possible they even filled in the survey together, which may further reduce heterogeneity and could create a social desirability bias. However, our study sample was similar to the general Dutch population aged ≥65 years [24] in terms of gender (66% female in both the general population and in our study sample) and age distribution (57% aged 65-75 years and 43% aged >75 years in the general population vs 56.2%, 59/105 and 43.8%, 46/105, respectively, in our study sample), indicating that our sample is at least broadly representative of seniors in the Netherlands.

Our sample could be affected by a participation bias: seniors who are willing to fill in an online survey may also be more willing to search for health information online compared to seniors who are capable of using the Internet but prefer not to fill in an online survey. Neither health information seeking nor use of the Internet as a health information resource were associated with health status in our study. However, people in poorer health may be less inclined to complete a survey and, thus, may be underrepresented in this study. All our respondents are connected to a senior organization, which may represent a more proactive group of seniors than the general population. Seniors who are more willing to participate in a survey may also be more willing to participate in their own health care decisions. We used the API to assess the respondents’ general desire for health information and involvement in health decisions. Our respondents had a median Decision Making scale score of 58 and a median Information Needs scale score of 71. The authors of the original API study reported a similar mean Information Needs scale score (79.5) to that found in our study, but a considerably lower Decision Making scale score (33.2) [16]. The authors also reported that the scores in their study tended to decrease with age, implying that we should expect lower scores in our older study population. The fact that our respondents seem to have a rather high desire for involvement in decision making could explain why we did not see an association between the API and information-seeking behavior. Finally, neither the original surveys on which our questions are based nor this survey have been validated, with the exception of the API, and all questions needed to be translated to the Dutch language. We cannot be sure that our respondents interpreted the questions in the way we intended. However, we used forward-and-back translation to retain comparability to the original studies.

### Comparison to Prior Work

The original US survey also reported a much higher use of paper-based resources (books and magazines) [8], whereas in our study we found that these resources were primarily used by people who also use the Internet for health information. This implies that offering both Internet-based and paper-based resources may reach the same audience twice rather than different audiences and, therefore, may miss an important segment of the population that uses neither. Use of health professionals, pharmacists, leaflets, telephone help lines, television, and radio were similar in groups with both high and low Internet use. In retrospect, these resources are more verbal (except leaflets) than the other resources, suggesting that differences in health literacy or preferred modes of communication may be in play. Apart from telephone help lines, they also require less active information-seeking behavior on the part of the patient, implying that ease of access could be a factor. Trust of information sources could also be a factor, although overall trust in both the Internet and in other sources of health information was higher for those who report higher use of the Internet (*P*<.001).We found that use and trust of information sources were correlated, but it is not clear if sources are not trusted because they are unfamiliar (not used) or if they are not used because they are not trusted. In conjunction with the finding that information seeking typically occurs after an appointment, the timing of information seeking may also play a role in the number and type of resources used. Because of prior experience with other health information resources, seniors may also be more inclined to use the Internet in conjunction with rather than instead of these other resources. Further research is needed to learn whether this finding persists in a larger sample including non-Internet users, and whether the aforementioned underlying factors can be correlated to the result or if the result can be explained by a theoretical model (eg, the Health Belief Model [25]). If this finding persists in a general population, it has important implications for theory and practitioners who may seek out better ways of helping seniors attain health information.

In the survey conducted in the general population in Germany, the rates of seeking information after an appointment (66.2%), to prepare for an appointment (53.8%), and in deciding whether to consult with a health professional (65%) were similar and in all cases higher than in our survey [4]. Notably, our respondents reported seeking information primarily after an appointment. Presumably, this is because patients hear new information during the appointment that, in turn, stimulates information-seeking behavior. Furthermore, patients who sought information after an appointment were more inclined to use the Internet for this search (29/37, 78% vs 10/16, 62% when preparing for an appointment and 8/18, 44% when deciding whether they should go to the doctor). Thus, there may be a relationship between the timing of information seeking and the choice of resource. This is a useful finding for those who hope to provide tools to help older patients prepare for a doctor’s visit because it implies that most potential users are unlikely to find the tool on their own before an appointment, but will need to be contacted in some other way.

The source of information varied substantially by clinical subject. The Internet was used most often when seeking information about symptoms, prognosis, and treatment options, but health professionals were asked for additional information about prescription medications, side effects, practical care information, and nutrition/exercise advice. The only subject for which paper-based resources were commonly reported was information about side effects, although the respondents may have been referring to reading the paper inserts that come with prescription medications. The 2005 US study reported that the Internet was commonly used for information about medications and nutrition and exercise [8], and a 2007 survey of US cancer patients found that information on treatment options, prognosis, and side effects were sought more on the Internet than information on coping with the disease [13], but neither study compared the Internet to other resources. The use of different resources for different subjects could be because of the availability of information from the Internet or other sources, a need for personalized information, a sense that some types of information on the Internet are more reliable than others, or other factors. Additional study is needed to learn whether this difference in use is attributable to preference or simple practicality.

As we reported previously [26], respondents reported both positive and negative emotional responses to the health information they found. The effects were not different between those who did and did not use the Internet for health information after correction for increased seeking of health information in general. Also, nearly all respondents expected their doctor to provide all necessary health information. This is a positive finding in that our respondents clearly trust their health care professionals, but it also places tremendous and possibly unrealistic expectations of the capacity to convey information in a typical 10-minute appointment. This suggests that all health information sought online and elsewhere is viewed as ancillary information by our respondents. This finding casts some doubt on whether patients would rely on a computer-based tool, such as a self-management program, to be a necessary part of their care.

Future study of seniors who use the Internet for (additional) health information should further investigate why and how seniors search for information on the Internet (eg, a preference for search engines vs health portals or whether the device used affects the search strategy [27]), whether they feel they have found the information they need, and whether they are able to understand and correctly judge the quality and reliability of information they find. A recent study of seniors asked to interpret a set of symptoms using online tools found that many of the participants had difficulty navigating the online tools and that this difficulty was correlated with reaching an incorrect conclusion [28]. Further study is also needed of seniors who do not use the Internet for health information. There are 2 groups of interest here: those who do seek health information but from other sources and those who do not actively seek health information. The latter group likely consists of people with little need for health information (healthy seniors or seniors in a stable health state) and people who have a need for health information but are not aware of it or prefer not to think about it. This latter population likely includes disadvantaged groups who are at higher risk for many health problems. Learning how to efficiently apply resources to reach those who actively seek information also frees more resources for reaching those who do not.

### Conclusions

We surveyed 118 seniors who use the Internet to learn how they use the Internet compared with other sources in searching for health information. Respondents used the Internet for health information as much as they asked health care professionals and pharmacists. Seniors who use the Internet for health information also report higher use of other sources of health information, particularly paper-based resources. This may imply that supplying both Internet and paper information is redundant and other channels must be used to reach those who will not find the information on the Internet. Most respondents who had searched for health information in the last year did so after an appointment, whereas only approximately half as many said they searched for information to decide to go to the doctor or prepare to go to the doctor. This implies that additional effort may be needed to encourage accessing information intended to prepare patients for an appointment. The resources used varied by health topic, implying that different channels may be preferred for different kinds of information. Although the findings of this survey should be considered preliminary, seniors who seek health information seem likely to use the Internet and seniors who do not use the Internet for health information also tend to make less use of health information resources apart from health care professionals. Future research should investigate how seniors seek and understand information on the Internet, whether seniors who seek information from all sources tend to be Internet users, and how to reach seniors who prefer not to use the Internet for health information.

## Multimedia Appendix 1

Survey instrument, with references given for the source of each item in the survey.

## Multimedia Appendix 2

Statistical results for association between age and gender and the timing and subject of information seeking. Estimates and confidence intervals for each analysis are reported.

## Abbreviations

API: Autonomy Preference Index
FDR: false discovery rate
SES: socioeconomic status
