# Supplementary material for: Health Information–Seeking Behavior of Seniors Who Use the Internet: A Survey
Source: J Med Internet Res. 2015 Jan 8;17(1):e10. doi: 10.2196/jmir.3749 (PMC4296102; doi:10.2196/jmir.3749)
Supplement: Supplementary file 1 [file jmir_v17i1e10_app1.pdf]

As a patient you can choose the care that best fits your personal situation, but only if you have the right information to make a good decision. The purpose of this survey is to learn how seniors and their caregivers stay informed about their health – both general health information (such as tips for staying healthy), and how you look for answers to specific questions or information about health problems. Then we will know how to best reach you and other seniors with important information about health.

Section 1: demographics and general health
Your information is analyzed anonymously and cannot be used to identify individuals.

	Year of birth	__________
	Gender	M | F
	Postcode (numbers only)	__________
	Highest education level	primary school | high school | vocational/housekeeping | associate's degree | bachelor | university/master
	Country of birth	__________
	Marital status	married | divorced | widowed | cohabitating | single
	Housing status	cohabitating (partner | friend | family) | living alone | nursing home
[D]	Are you the primary caretaker for someone with a serious or chronic health condition?	yes | no
[I]	In general, how is your health?	very good | good | fair | bad | very bad
[E]	I can make an appointment for routine care (e.g. with my primary care doctor) as soon as I want	strongly agree | agree | disagree | strongly disagree

[E] Rating of all health care received in the last 12 months:
1.....2.....3.....4.....5.....5.....6.....7.....8.....10
unacceptable....................average...........................outstanding

Section 2: API
The questions for the Autonomy Preference Index were drawn from Simon et al.[J]*, which were in turn based on Ende et al.*[K]

Section 3: Sources of health information
Health information is information about preventative care (such as vaccinations or good nutrition), specific conditions (such as diabetes or cancer), and medications or other forms of treatment. This section is about the ways you stay informed about your health.

[D] **How much of your health information do you get from each of the following sources:**

[D,I]	direct contact with a health professional	a lot | some | a little | none
[D,I]	pharmacy	a lot | some | a little | none
[C]	leaflets at the doctor's office (such as in the waiting room)	a lot | some | a little | none
[C]	telephone help line	a lot | some | a little | none
[D,I]	television	a lot | some | a little | none
[D,I]	radio	a lot | some | a little | none
[I]	newspapers	a lot | some | a little | none
[B]	magazines specifically about health	a lot | some | a little | none
[D,I]	other magazines	a lot | some | a little | none
[D,I]	friends /family	a lot | some | a little | none
[N]	church/religious group	a lot | some | a little | none
[I]	courses and lectures	a lot | some | a little | none
[D,I]	internet	a lot | some | a little | none
[H]	self-help/patient groups	a lot | some | a little | none
[D,I]	books/encyclopedias	a lot | some | a little | none

[N]	the library	a lot | some | a little | none
[N]	other/comments	

[D] **Please indicate how much you *trust* health information from each of the following sources[1]:**

[D,I]	direct contact with a health professional	a lot | some | a little | none
[D,I]	pharmacy	a lot | some | a little | none
[C]	leaflets at the doctor's office (such as in the waiting room)	a lot | some | a little | none
[C]	telephone help line	a lot | some | a little | none
[D,I]	television	a lot | some | a little | none
[D,I]	radio	a lot | some | a little | none
[I]	newspapers	a lot | some | a little | none
[B]	magazines specifically about health	a lot | some | a little | none
[D,I]	other magazines	a lot | some | a little | none
[D,I]	friends /family	a lot | some | a little | none
[N]	church/religious group	a lot | some | a little | none
[I]	courses and lectures	a lot | some | a little | none
[D,I]	internet	a lot | some | a little | none
[H]	self-help/patient groups	a lot | some | a little | none
[D,I]	books/encyclopedias	a lot | some | a little | none
[N]	the library	a lot | some | a little | none
[N]	other/comments	

Section 4: Searching for health information

People often have questions about health. This section is about searching for information beyond what your doctor provides to you. You may have asked a health professional for more information, or asked someone other than a doctor (such as family, friends, classes, or a patient support group). You may have searched on the internet, or looked for written information (such as in magazines or at the library). Please tell us if you have *sought* additional information, even if you didn't find what you were looking for. Check all that apply.

[D] **In the last 12 months, I have sought health information**:

[H,I]	to decide whether I need to see a doctor	no | yes [If yes, how?: asked a health professional | asked other people | on the internet | written materials]
[H,I]	to prepare for an appointment	no | yes [If yes, how?: asked a health professional | asked other people | on the internet | written materials]
[H,I]	to look up information after an appointment	no | yes [If yes, how?: asked a health professional | asked other people | on the internet | written materials]

[D] **I have sought health information about**:

[D1]	specific symptoms, to find out what might be causing them	no | yes [If yes, how?: asked a health professional | asked other people | on the internet | written materials]
[G]	prognosis	no | yes [If yes, how?: asked a health professional | asked other people | on the internet | written materials]
[G]	treatment options	no | yes [If yes, how?: asked a health professional | asked other people | on the internet | written materials]
[D]	prescription drugs	no | yes [If yes, how?: asked a health professional | asked other people | on the internet | written materials]
[G]	side effects of treatment or medication	no | yes [If yes, how?: asked a health professional | asked other people | on the internet | written materials]
[G]	coping with a disease	no | yes [If yes, how?: asked a health professional | asked other

[1] Respondents commented that the phrasing of this question did not distinguish between "I don't use this source because I don't trust it" and "I don't know if I trust this source, because I don't use it." The authors suggest adding a "not applicable" option to these questions.

	people | on the internet | written materials]
[A] practical care information (e.g. bathing, first aid, etc.)	no | yes [If yes, how?: asked a health professional | asked other people | on the internet | written materials]
[B,D] nutrition/physical exercise	no | yes [If yes, how?: asked a health professional | asked other people | on the internet | written materials]

[N] If I have a need for information, I prefer to (please choose 1 option):	I don't search for health-related information<br>Ask a health professional<br>Search on the internet<br>Search using sources other than the internet (magazines, friends, etc.)
[F] I often want more health information but don't know where to find it	strongly agree | agree | disagree | strongly disagree
[C] I expect my doctor/health professionals to provide me with all of the information that I need.	strongly agree | agree | disagree | strongly disagree
[E] I have had difficulty finding health information in my primary language	never | once | sometimes | often

Section 5: Consequences of health information seeking (Results previously published in [L])

[I] **Has the health-related information you found led to**:

[D]	deciding *to see* a doctor	never | once | sometimes | often
[H]	deciding *not to see* a doctor	never | once | sometimes | often
[D]	a conversation with a doctor about what I found	never | once | sometimes | often
[H,I]	willingness to change diet/lifestyle habits	never | once | sometimes | often
[H,I]	change of medicine without consulting a health professional	never | once | sometimes | often
[H,I]	feelings of anxiety	never | once | sometimes | often
[H,I]	feelings of reassurance or relief	never | once | sometimes | often
[H,I]	suggestions/queries about a diagnosis	never | once | sometimes | often
[H]	suggestions/queries about a treatment	never | once | sometimes | often
[E]	more knowledge and understanding of a specific condition, disease or treatment	never | once | sometimes | often
[N]	feeling more confused about a specific condition, disease, or treatment	never | once | sometimes | often

other/comment____

[] Someone helped me with filling in this questionnaire

References

A. Kernisan LP1, Sudore RL, Knight SJ. Information-seeking at a caregiving website: a qualitative analysis. J Med Internet Res. 2010 Jul 28;12(3):e31. doi: 10.2196/jmir.1548.
B. Taha J, Sharit J, Czaja S. Use of and satisfaction with sources of health information among older Internet users and nonusers. Gerontologist 2009: 49(5): 663-73.

C. Rokade A, Kapoor PK, Rao S, Rokade V, Reddy KT, Kumar BN. Has the internet overtaken other traditional sources of health information? Questionnaire survey of patients attending ENT outpatient clinics. Clin Otolaryngol Allied Sci. 2002 Dec;27(6):526-8.

D. Rideout VJ, Neuman T, Kitchman M, Brodie M, The Kaiser Foundation. E-health and the elderly: How seniors use the internet for health in-formation: Key findings from a national survey of older Americans. 2005
D1. modified from (D)

E. Zhang, Y, Jones B, Spalding M, Young R, Ragain M. Use of the internet for health information among primary care patients in rural West Texas. South Med J, 2009. 102(6): p. 595-601.
Questions in this study were derived from Baker L, Wagner TH, Singer S, et al. Use of the Internet and e-mail for health care information: results from a national survey. *JAMA* 2003;289:2400 –2406.

F. Ybarra, ML, Suman M. Help seeking behavior and the Internet: a national survey. Int J Med Inform, 2006. 75(1): p. 29-41.

G. Castleton K, Fong T, Wang-Gillam A, Waqar MA, Jeffe DB, Kehlenbrink L, Gao F, Govindan R. A survey of Internet utilization among patients with cancer. Support Care Cancer. 2011 Aug;19(8):1183-90. doi: 10.1007/s00520-010-0935-5.

H. Dumitru RC, Bürkle T, Potapov S, Lausen B, Wiese B, Prokosch HU. Use and perception of internet for health related purposes in Germany: results of a national survey. Int J Public Health, 2007. 52(5): p. 275-85.

I. Andreassen HK1, Bujnowska-Fedak MM, Chronaki CE, Dumitru RC, Pudule I, Santana S, Voss H, Wynn R. J. European citizens' use of E-health services: a study of seven countries. BMC Public Health. 2007 Apr 10;7:53.

J. Simon D1, Kriston L, Loh A, Spies C, Scheibler F, Wills C, Härter M. Confirmatory factor analysis and recommendations for improvement of the Autonomy-Preference-Index (API). Health Expect. 2010 Sep;13(3):234-43. doi: 10.1111/j.1369-7625.2009.00584.x.

K. Ende J1, Kazis L, Ash A, Moskowitz MA. Measuring patients' desire for autonomy: decision making and information-seeking preferences among medical patients. J Gen Intern Med. 1989 Jan-Feb;4(1):23-30.

L. Medlock S, Eslami S, Askari M, Sent D, de Rooij SE, Abu-Hanna A. The consequences of seniors seeking health information using the internet and other sources. Stud Health Technol Inform. 2013;192:457-60.

N. new
