# Supplementary material for: Health Information–Seeking Behavior of Seniors Who Use the Internet: A Survey
Source: J Med Internet Res. 2015 Jan 8;17(1):e10. doi: 10.2196/jmir.3749 (PMC4296102; doi:10.2196/jmir.3749)
Supplement: Supplementary file 2 [file jmir_v17i1e10_app2.pdf]

**Association of age with seeking information after an appointment, before an appointment (to prepare for an appointment), or to decide whether an appointment is needed.**

All Confidence Intervals (CI) are 95%.

age::seeking information after an appointment
> lm(types$age ~ types$after)
β =  -2.5539
*P* = 0.0933
CI = -5.544627  0.4367357

age::seeking information to prepare for an appointment
> lm(types$age ~ types$before)
β =  -0.5641
*P* = 0.745
CI = -4.000424  2.872164

age::deciding whether to go to the doctor
> lm(types$age ~ types$decide)
β =  0.5023
*P* = 0.793
CI = -3.281429  4.28608

**Association of age with searching for types (topics) of health information**

age::searching for any type of health information
>lm(types$age ~ types$anytype)
β =  0.6035
*P* = 0.701
CI = -2.499566  3.706516

age::information on treatment
> lm(types$age ~ types$tx)
β =  -1.509
*P* = 0.329
CI =   -4.562831  1.545637

age::information on side effects
> lm(types$age ~ types$bijwerk)
β =  2.6626
*P* = 0.076
CI =  -0.2841205  5.609373

age::information on coping with illness
> lm(types$age ~ types$coping)
β =  0.7660
*P* = 0.64
CI =  -2.47648  4.008566

age::prescription medications
> lm(types$age ~ types$rx)
$\beta$ = 2.7190
$P$ = 0.0754
CI = -0.2832506 5.721169

age::practical care information
> lm(types$age ~ types$practical)
$\beta$ = 1.5635
$P$ = 0.434
CI = -2.385616 5.51260

age::prognosis
> lm(types$age ~ types$px)
$\beta$ = -1.0471
$P$ = 0.514
CI = -4.21718 2.123062

age::symptoms
> lm(types$age ~ types$symptoms)
$\beta$ = 0.9112
$P$ = 0.549
CI = -2.094967 3.91737

age::nutrition and exercise
> lm(types$age ~ types$voeding)
$\beta$ = 0.1341
$P$ = 0.931
CI = -2.953408 3.221662

Use FDR correction for multiple testing on relationship between age and topics:
> psAge <- c(0.3295, 0.07604, 0.6403, 0.07539, 0.434, 0.5139, 0.5491, 0.9315)
> p.adjust(psAge, method = "fdr")
0.7317714
0.3041600
0.7317714
0.3041600
0.7317714
0.7317714
0.7317714
0.9315000

**Association of gender with seeking information after an appointment, before an appointment (to prepare for an appointment), or to decide whether an appointment is needed.**
gender::seeking information after an appointment
> glm(types$gender ~ types$after)
$\beta$ = -0.25700
P = 0.00968

CI = -0.4479623 -0.06603064

gender::seeking information to prepare for an appointment
> glm(types$gender ~ types$before)
β =  -0.02676
P = 0.823
CI = -0.2606230 0.2071113

gender::deciding whether to go to the doctor
> glm(types$gender ~ types$decide)
β =  -0.06429
P = 0.608
CI = -0.3090321 0.1804606

**Association of gender with searching for types (subjects) of health information**

gender::searching for any type of health information
> glm(types$gender ~ types$anytype)
β =  -0.31429
P = 0.00202
CI =  -0.5087421 -0.1198293

The following results include the variable (anytype) to adjust for the fact that more women than men had searched for health information in general in the last 12 months.

gender::information on treatment
> glm(types$gender ~ types$tx + types$anytype)
β =   -0.09033
P = 0.4473
CI =   -0.5325412 -0.04658339

gender::information on side effects
> glm(types$gender ~ types$bijwerk + types$anytype)
β =   0.01515
P = 0.90172
CI =  -0.5123242 -0.06267577

gender::information on coping with illness
> glm(types$gender ~ types$coping + types$anytype)
β = -0.15758
P = 0.1693
CI =  -2.47648  4.008566

gender::prescription medications
> glm(types$gender ~ types$rx + types$anytype)
β =  0.21860
P = 0.06610
CI =  -0.74001203 -0.2539274

gender::practical care information

> glm(types$gender ~ types$practical + types$anytype)
β = -0.15033
P = 0.24759
CI =  -0.5308460 -0.1179954

gender::prognosis
> glm(types$gender ~ types$px + types$anytype)
β =  -0.09477
P = 0.40632
CI =  -0.5325546 -0.08360705

gender::symptoms
> glm(types$gender ~ types$symptoms + types$anytype)
β =  -0.02174
P = 0.8594
CI =  -0.5615242 -0.05710934

gender::nutrition and exercise
> glm(types$gender ~ types$voeding + types$anytype)
β =  -0.10000
P = 0.3870
CI =  -0.5248234 -0.05017659

Use FDR correction for multiple testing on relationship between gender and topics:
> psGen <- c(0.4473,0.90172 ,0.1693,0.06610,0.24759,0.40632,0.8594,0.3870)
> p.adjust(psGen, method = "fdr")
0.59640
0.90172
0.59640
0.52880
0.59640
0.59640
0.90172
0.59640
